# State-of-the-Art Virology Research in Norway

**DOI:** 10.3390/v15122383

**Published:** 2023-12-05

**Authors:** Christine Hanssen Rinaldo, Morten Tryland

**Affiliations:** 1 Department of Microbiology and Infection Control, University Hospital of North Norway, N-9038 Tromsø, Norway; 2 Metabolic and Renal Research Group, Department of Clinical Medicine, UiT The Arctic University of Norway, N-9037 Tromsø, Norway; 3 Department of Forestry and Wildlife Management, Faculty of Applied Ecology, Agricultural Sciences and Biotechnology, Inland Norway University of Applied Sciences, N-2480 Koppang, Norway; 4 Arctic and Marine Biology, UiT The Arctic University of Norway, N-9037 Tromsø, Norway

## 1. Introduction

Norway is situated in a remote and sparsely inhabited part of the world with about 5.4 million human inhabitants spread over 385,207 km^2^ of forest, tundra, coastal landscapes and mountains. The conditions are generally conducive to good health for humans, as well as domesticated, wild, land and marine animals and plants. For instance, until recently, Norway was not affected by highly pathogenic avian influenza (HPAI). However, in 2020, HPAI A/H5N8 was detected in wild birds in south Norway for the first time [1,2], and since then, HPAI A/H5N5 and A/H5N1 have been detected in commercial poultry and wild birds, with a large outbreak in seabirds in northern Norway during summer 2023 [3]. HPAI A/H5N1 has also been detected in red foxes [2]. Furthermore, in 2022, Newcastle disease virus, which had not been detected in Norway since 2003, was detected in wild pigeons in Oslo and lead to the first outbreak of Newcastle disease on a commercial poultry farm [4]. These findings are strong reminders of the fact that ecosystems are changing, affecting the distribution of pathogens and facilitating zoonoses and emerging diseases.

The goal of this Special Issue was to present the state-of-the-art work of virology researchers in Norway working on viruses that infect humans, animals, fish, arthropods, plants and bacteria. In total, 10 papers were accepted between August 2022 and February 2023. During that time, COVID-19 was still a Public Health Emergency of International Concern (PHEIC). As expected, the topics of the recently submitted papers were strongly influenced by the ongoing COVID-19 pandemic. Since the 5th of May 2023, COVID-19 is no longer a PHEIC, but it is an important ongoing health issue (https://www.who.int/europe/news/item/05-05-2023-statement-on-the-fifteenth-meeting-of-the-international-health-regulations-(2005)-emergency-committee-regarding-the-coronavirus-disease-(COVID-19)-pandemic, accessed on 4 December 2023). The purpose of this Special Issue is to encourage readers to read the papers presented.

## 2. An Overview of Published Papers

Ertesvåg et al. (contribution 1) provided a case report of a 59-year-old patient receiving the B cell depletion therapy rituximab for relapsing–remitting multiple sclerosis and who then became infected with the SARS-CoV-2 Delta variant. The patient developed a prolonged infection with fever lasting more than 90 days, and was hospitalized thrice. The patient was treated with several antiviral drugs, but only fully recovered 6 months post-rituximab infusion, when rituximab’s effect subsided. Of note, the patient had received the SARS-CoV-2 mRNA vaccine while under bi-annual rituximab treatment. The authors concluded that B cells may be essential to clear SARS-CoV-2 infection, and therefore, that longer intervals in the rituximab treatment should be considered. They also recommended that for patients with immune dysregulation, PCR analysis of bronchoalveolar lavage and serum should be performed to detect low-grade SARS-CoV-2 replication. As rituximab, which was originally developed as a treatment for lymphoma, is now increasingly used also as a treatment for autoimmune diseases, it is important to communicate the above findings to attending physicians.

Moen et al. (contribution 2) investigated AY.63, a sub-lineage of the SARS-CoV-2 Delta variant first registered in Norway on the 10th of June 2021, and then, rapidly became the dominating variant in Norway. Compared to B.1.617.2, which was first detected in India in October 2020, AY.63 had a spike mutation (A222V) and a deletion in the nsp1 gene, but this did not cause increased fitness. Interestingly, AY.63 was almost exclusively found in Norway. The authors concluded that the success of AY.63 was due to its introduction taking place at a time during which there were low circulating levels of SARS-CoV-2 and relaxation of contact-reducing regulations. By the end of November 2021 AY.63 became extinct in Norway, probably because domestic transmission alone during a period of public health measures could not sustain its circulation. This study clearly shows that other factors than viral fitness influence the circulation of SARS-CoV-2 variants. Worldwide, the Omicron variant has replaced the Delta variant.

Granerud et al. (contribution 3) investigated whether the SARS-CoV-2 Omicron variant generated a higher viral load than the Delta variant by investigating nasopharyngeal swabs and saliva from 52 Omicron cases and 17 Delta cases about 7 and 14 days after symptom onset. All samples were analyzed via real-time PCR, which included the WHO international standard, and the viral copy number was normalized to the amount of human DNA (1000 cells), presumably to minimize the effect of different sampling efficiencies. The authors concluded that Omicron cases carried a higher viral load and showed more sustained viral shedding compared to the Delta cases, particularly in the nasopharynx. Furthermore, the authors reported that the nasopharynx contains higher and more lasting viral loads than saliva, and therefore, is a better specimen for SARS-CoV-2 testing, which is important information for diagnostic laboratories despite the less invasive sampling of saliva. 

Kittang et al. (contribution 4) compared the incidence of previous cases of COVID-19 among 747 healthcare workers (HCWs) in Norwegian nursing homes or hospitals, including municipal emergency rooms, from the end of September 2020 to January 2021, i.e., just before the introduction of the SARS-CoV-2 vaccine. The diagnosis was based on self-reported PCR positivity and serological evidence of SARS-CoV-2 infection. Anti-spike protein antibodies were investigated by two previously described in house assays [5]. No significant difference in positivity was found (4% versus 6%). Of note, 50% of nursing home HCWs exposed to COVID-19 patients did not use full personal protective equipment (PPE). One probable reason for this was the poor availability of PPE in nursing homes in Norway in the first phase of the pandemic. Importantly, the risk for COVID-19 among HCWs in high-risk departments was not significantly higher than for HCWs without occupational exposure, showing that HCWs can work safely with COVID-19 patients. 

Syre et al. (contribution 5) compared three commercial anti-SARS-CoV-2 serological assays (the Wantai receptor binding domain (RBD) total antibody assay, the Liaison S1/S2 IgG assay and the Alinity i nucleocapsid IgG assay), using the in house assays used by Kittang et al. In total, 563 HCWs and patients were included, and of these, 207 cases were SARS-CoV-2 PCR-positive (37%), 15 were PCR-negative and 314 had no PCR test. The study took place from March 2020 to June 2021. For 46 SARS-CoV-2-positive cases, sera were sampled at more than one time point following PCR positivity. The authors concluded that the different tests showed variation in sensitivity but that specificities were generally high. Importantly, the study showed that the time of sampling following PCR positivity greatly impacted test performance. The Wantai RBD total antibody test performed best in the first 39 days following PCR positivity. For all tests, the sensitivity was higher in sera sampled one to two months after a positive PCR test compared to those sampled in the first weeks following PCR positivity. 

Augustinussen et al. (contribution 6) compared anti-spike protein IgG levels in 82 HCWs through three different SARS-CoV-2 vaccine regimens involving the recombinant adenovirus-based vaccine ChAdOx1 nCov-19 in combination with an RNA vaccine (either BNT162b2 produced by Pfizer-BioNTech or mRNA-1273 produced by Moderna) or only mRNA vaccines, where the first two doses were given three or six weeks apart. In order to detect unnoticed SARS-CoV-2 infection, all samples were also analyzed for anti-nucleocapsid IgG. Finally, the effect of post-vaccination infection on anti-spike protein IgG persistence was investigated. The authors concluded that despite initial differences, all three vaccination regimes elicited similar levels of anti-spike protein IgG after the third dose, and that the levels were higher than after only one or two doses. However, the levels of anti-spike IgG declined significantly in all groups during the following six months. Importantly, post-vaccination infection seemed to increase the persistence of anti-spike protein IgG. As HCWs were mainly given vaccines based on availability and not based on the desire for a particular vaccine, the fact that three doses of different vaccines regimens give similar IgG levels is encouraging. 

Coronaviruses not only cause disease in humans, but are also major pathogens in cattle, impacting animal welfare and causing economic losses. Shakya el al. (contribution 7) established bovine enteroids as an authentic in vitro replication system for bovine coronavirus (BCoV). After characterization of the enteroid cell population, the expression of selected immune genes during BCoV infection was compared to the expression of immune genes in a previously used 2-D model of HCT-8 cells. The enteroids were permissive for BCoV, but the gene expression demonstrated a host response that was different from that reported for BCoV-infected HCT-8 cells. The authors concluded that further studies are needed to clarify whether enteroids represent a good model for the bovine intestine.

Hepatitis E virus (HEV) is a major cause of acute hepatitis. In Asia and Africa, large outbreaks are seen, and HEV infection particularly affects pregnant women and their offspring. In industrialized countries, other viral genotypes cause sporadic cases of chronic hepatitis in immunocompromised individuals, and neurologic disorders in previously healthy individuals [6]. In Norway, seroprevalence studies of blood donors and of the general adult population have found anti-HEV IgG seroprevalences of about 14% and 11.4%, respectively [7,8]. Sero-surveillance studies in nonindustrial countries have been hampered by a lack of infrastructure and funding. In the 1990s, dried blood spots (DBS) on Guthrie cards were used for the first time to estimate the prevalence of HIV infection in childbearing women [9]. Øverbø et al. (contribution 8) optimized the Wantai HEV IgG ELISA for DBS and compared the result of 300 pairs of DBS and plasma/serum samples from 150 participants in an HEV vaccine study, originating from rural areas in Bangladesh. DBS were prepared with capillary blood directly collected via fingerprick or from whole blood collected via venepuncture. The authors found that anti-HEV IgG stored in DBS was stable and that there was a strong correlation between the analyses of DBS and plasma/serum samples. The study suggests that DBS can be used for serological studies, particularly in economic-resource-limited areas. As there seems to be a positive correlation between IgG titer and sensitivity, DBS seems to work well for HEV vaccine studies where high antibody titers are expected. However, for HEV seroprevalence studies where great variation in the anti-HEV IgG titer is expected, the use of DBS needs further optimization. 

Norway hosts about 25,000 wild reindeer (*Rangifer t. tarandus*) living in 24 more-or-less separate populations. In addition, there are about 220,000 semi-domesticated reindeer of the same subspecies, most of them associated with the Saami reindeer herding in the northern part of the country. In 1787, 35 semi-domesticated Norwegian reindeer were exported from Norway to Iceland, and these animals represent the origin of about 5000 wild Eurasian tundra reindeer living in Iceland today. Tryland et al. (contribution 9) looked for antibodies against alphaherpesvirus, gammaherpesvirus, pestivirus, bluetongue virus and Schmallenberg virus (n = 281). While most reindeer were found to be seronegative for these viruses, two animals had antibodies against pestivirus and two had antibodies against gammaherpesvirus (malignant catarrhal fever group, MCFV). The seroprevalence for these two viruses seems to be very low and their clinical impact needs further investigation. They also detected parapoxvirus-specific DNA in nose swabs from two animals, indicating that Orf virus (ORFV) is circulating, probably as a spill-over infection from sympatric sheep. Interestingly, the authors found no evidence of alphaherpesvirus infections in Icelandic reindeer, a virus (i.e., CvHV2) that seems to be enzootic in most investigated reindeer and caribou populations in the northern hemisphere [10].

In addition to traditional livestock and meat production, farmed fish have become the most important production species in Norway, with about 1.5 million tons of salmon and rainbow trout produced annually. Viral infections are one of the major threats in the production of these fish [11]. One of the viruses infecting farmed as well as wild fish is Viral hemorrhagic septicemia virus (VHSV). Bergh et al. (contribution 10) performed a challenge experiment using two VHSV isolates from herring from the western coast of Norway and Denmark, respectively, and herring from the west coast of Denmark. The herring were susceptible to both strains of VHSV, but infection with the Norwegian strain (genotype Ib) had higher prevalence and a mortality rate of 47% compared to the Danish strain (genotype III), which had a mortality rate of 6%. The authors conclude that genotype Ib may have negative impacts on wild Atlantic herring stocks, and that further investigations of VHSV infection of wild herring are needed. 

## 3. Concluding Remarks

With humans’ travelling habits and the seasonal migrations and altered distribution of birds, mammals, fish and insect vectors, and also due to the ecological impacts of climate change and anthropogenic encroachment, Norway will be exposed to new viral pathogens, new vectors and altered patterns of infection biology and epidemiology, of which the COVID-19 pandemic and the emergence and spread of HPAI are recent examples. Thus, high-quality virus research addressing transdisciplinary global and regional challenges is more important than ever.

We would like to thank all of the authors who contributed to this Special Issue of *Viruses*, and believe that this collection represents a cross-section of the current virus research in Norway.
